# Smartphone-based inertial measurements during Chester step test as a predictor of length of hospital stay in abdominopelvic cancer postoperative period: a prospective cohort study

**DOI:** 10.1186/s12957-024-03337-1

**Published:** 2024-02-28

**Authors:** Ananda Quaresma Nascimento, Letícia Akemi Rosa Nagata, Manuela Trindade Almeida, Vanessa Ladyanne da Silva Costa, Ana Beatriz Rocha de Marin, Victória Brioso Tavares, Geraldo Ishak, Bianca Callegari, Enzo Gabriel Rocha Santos, Givago da Silva Souza, João Simão de Melo Neto

**Affiliations:** 1 Federal University of Pará (UFPA), Belém, PA Brazil; 2 Institute of Exact and Natural Sciences, Federal University of Pará, Belém, Brazil; 3 Clinical and Experimental Research Unit of the Urogenital System (UPCEURG), Institute of Health Sciences of Federal University of Pará, Mundurucus street, Guamá, Belém, PA 4487CEP: 66073-000 Brazil

**Keywords:** Mobile applications, Exercise test, Length of stay

## Abstract

**Background:**

Objective assessment of pre-operative functional capacity in cancer patients using the smartphone gyroscope during the Chester step (CST) test may allow greater sensitivity of test results. This study has investigated whether the CST is a postoperative hospital permanence predictor in cancer patients undergoing abdominopelvic surgery through work, VO2MAX and gyroscopic movement analysis.

**Methods:**

Prospective, quantitative, descriptive and inferential observational cohort study. Fifty-one patients were evaluated using CST in conjunction with a smartphone gyroscope. Multivariate linear regression analysis was used to examine the predictive value of the CST.

**Results:**

The duration of hospital permanence 30 days after surgery was longer when patients who performed stage 1 showed lower RMS amplitude and higher peak power. The work increased as the test progressed in stage 3. High VO2MAX seemed to be a predictor of hospital permanence in those who completed levels 3 and 4 of the test.

**Conclusion:**

The use of the gyroscope was more accurate in detecting mobility changes, which predicted a less favorable result for those who met at level 1 of the CST. VO2MAX was a predictor of prolonged hospitalization from level 3 of the test. The work was less accurate to determine the patient's true functional capacity.

## Introduction

In patients with advanced or recurrent primary tumor in the pelvic or abdominal cavity, surgical resection is usually performed as a curative option that offers a greater chance of survival [1, 2]. However, extended surgery can lead to serious complications, disability, and higher health care costs, as well as prolonged hospitalization, morbidity, and mortality [3, 4]. Such outcomes are often attributed to a decrease in the preoperative physical capacity of the cancer patient, resulting from a reduced physiological reserve that predisposes the patient to an adverse postoperative period [5].

The cardiopulmonary exercise test (CPET) is considered the gold standard for assessing cardiorespiratory function because it can determine maximal oxygen consumption (VO2max) and predict surgical risk [6]. However, it requires specialized equipment, space, and trained personnel to perform, making it expensive and complex [7]. Therefore, field tests to determine exercise capacity, such as the 6-minute walk test (6MWT), incremental shuttle walk test (ISWT), and stair climbing test (SCT), are becoming more feasible and are recommended for preoperative physical examinations [8].

The Chester Step Test (CST) is a promising test for measuring cardiovascular fitness in cancer patients. It involves climbing up and down a 20-cm-high step at a set speed and monitoring the heart rate response to the exertion [9, 10]. This test has been previously validated to assess physical fitness in patients with chronic obstructive pulmonary disease (COPD) [11] and has been used in other contexts [12, 13] with high reliability in test-retest studies [10, 14]. The CST measures several components, including heart rate (HR), subjective exertion perception (RPE), estimated maximum heart rate for age (HRmax), and estimated oxygen consumption (VO2) of each test phase [10].

Several studies have used inertial sensors in the performance of these alternative physical assessment tests to capture additional characteristics related to the activity performed by the patient [15]. This approach provides a useful alternative for objective, discrete, and continuous acquisition of physical activity data at low cost and high practicality [16].

In the present study, a Chester Step Test protocol was applied and inertial displacements were recorded during performance of a submaximal step test in patients in the preoperative period of abdominopelvic cancer surgery. The aim was to determine whether preoperative inertial measurements during the Chester Step Test, as well as its results, can predict the length of hospital stay in the postoperative period.

## Materials and methods

### Ethical aspects

This research was carried out in accordance with the ethical principles established in Resolution 510 (07/04/2016) of the National Health Council (CNS) and was approved by the Ethics Committee for Research Involving Human Subjects of the João de Barros Barreto University Hospital of the Federal University of Pará (Report No. 5.282.029). All participants gave written informed consent after receiving a detailed explanation of the procedures.

### Study design

This is a prospective cohort observational study with quantitative, descriptive, and inferential analyses.

### Setting and period of the study

The study was conducted from May to October 2022 in the Surgical Clinic of the João de Barros Barreto University Hospital.

### Population

Cancer patients who had undergone abdominal or pelvic surgery participated in the study.

### Eligibility criteria

Male and female patients over 18 years of age with malignant neoplasms in the abdominal or pelvic region and who were scheduled to undergo surgery and had given written informed consent participated in the study. Patients who were unable to perform the simplest level of the Chester Step Test, who had motor or cognitive impairment, who had evidence of metastatic disease (due to the possibility of an increase in the number of surgical sites, which could interfere with the prognosis during the postoperative period), who had a history of unstable cardiac or pulmonary disease, or who were scheduled for repeat surgery were excluded from the study.

### Sampling

A nonprobabilistic intentional-type sample was used.

### Sample

The sample calculation was performed using the G*Power 3.1 application. The partial R^2^ used to calculate the minimum sample size for predicting CST was estimated in a pilot data collection, yielding an effect size f^2^ of 1.78. The sample size was calculated using the total number of predictors (RMS amplitude; frequency; peak power; work; VO2max; level) of 5 with 2 positive predictors; α err prob of 0.05; and β of 0.20. A required sample size of 11 subjects was calculated.

**Fig. 1:**
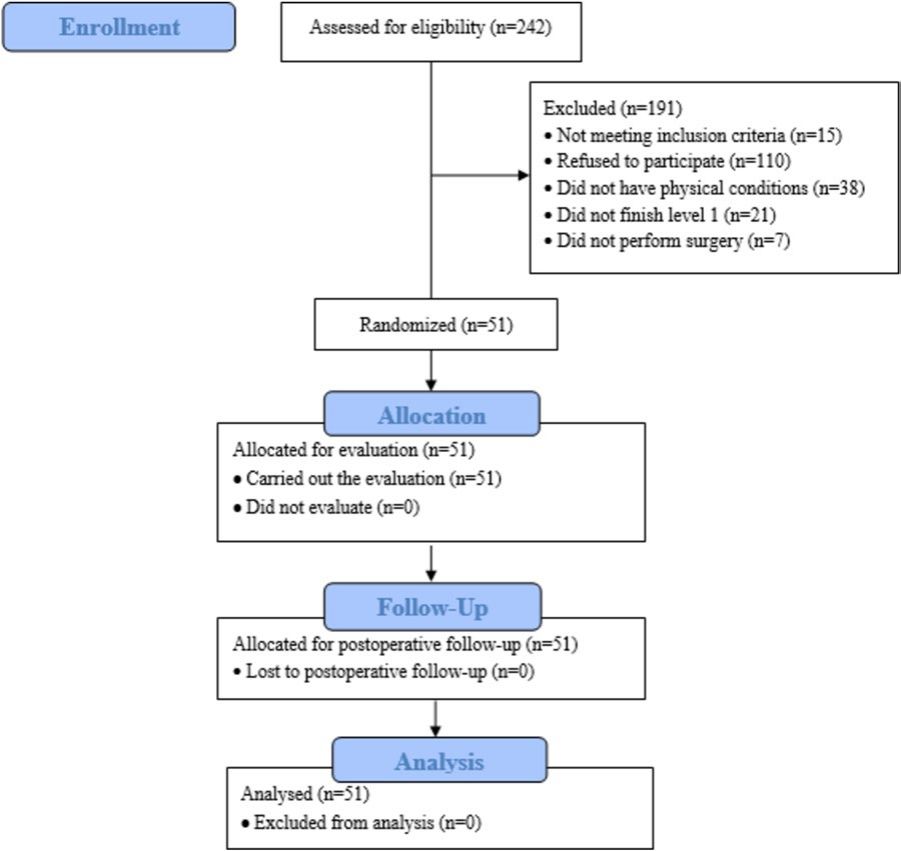
Flowchart for the selection of subjects

The original sample consisted of 242 patients, 51 of whom were selected according to the eligibility criteria (Fig 1).

### Data collection and variables

A form developed by the authors was used to collect social, anthropometric, and clinical data from all patients. The social variables included in the data collection were sex (female or male), age (years), skin color (white, brown, and black), education (primary, secondary, or higher education), marital status (single, married, and judicially separated or divorced), and family income (no income; < 1 minimum wage and > 1 minimum wage). The Brazilian minimum wage (R$1,212.00) was used as the minimum wage.

The anthropometric variables collected were weight (kg), height (m), and body mass index (BMI). Clinical variables collected included the presence of concomitant diseases such as hypertension, diabetes, heart disease, and obesity; current or past smoking and alcohol habits; main diagnostic hypotheses; surgeries performed; frequency of admission to the intensive care unit; need for invasive mechanical ventilation; average length of stay after surgery (days); oncological diagnosis (months); duration of surgery (hours); and frequency of deaths in the postoperative period.

All study participants had a smartphone attached to the patient's hip with a neoprene band at the level of the base of the sacrum. Participants were instructed to stand in front of a step (20 cm x 40 cm x 60 cm) and walk up and down in response to a voice command from the experimenter, following a series of beeps delivered by a fixed-beat digital metronome. The test consisted of 5 phases, each lasting 2 minutes, resulting in a total test time of 10 minutes. The speed of the metronome started at 15 steps/min and was increased by 5 steps/min every 2 minutes: stage 1 (15 steps/min), stage 2 (20 steps/min), stage 3 (25 steps/min), stage 4 (30 steps/min), stage 5 (35 steps/min) [11].

During the test, heart rate and oxygen saturation were recorded using a Polar frequency meter (POLAR® model H9) and pulse oximeter (G-TECH® model Oled Graph), respectively. The modified Borg Perception of Effort Scale [17] was used to assess participants' perception of dyspnea and lower limb fatigue at two time points: at rest and immediately after exercise. The test was terminated when the participant showed signs of fatigue, restrictive dyspnea, chest or leg pain, and exhaustion, or could not maintain stride rate for 15 seconds. The test was considered complete when the participant reached HR, which was 80% of his or her maximum predicted HR based on age (220 - age in years) [18], or when he or she reached level 5, which was the end of the 10-minute test.

To predict retention time, performance during the test was assessed using the following methods. First, the test score was determined by calculating the work performed, which was estimated using the following equation [step height (m) × total number of steps × weight (kg) × 0.16357] [19]. Second, VO2max was estimated using the Chester Step Test Calculator (Assist Creative Resources, Wrexham, UK) [20]. In addition, the mobile application Momentum Science (https://play.google.com/store/apps/details?id=com.beetsoftware.momentum_science) was used during the performance of the CST. The app used the smartphone's built-in triaxial gyroscope (model lsm6do, STMicro, acquisition rate: 20 Hz, 16 bits) built-in android smartphone (MOTOROLA® model Moto G9 Plus). The text files of the gyroscopic time series were imported into MATLAB/Octave routines. The X-, Y-, and Z-axis measurements were subjected to a detrending process and filtered using a second-order Butterworth filter after the zero-phase bandpass. A resulting vector was calculated from the three time series according to Eq. 1.

1 $$rv=\sqrt{{X}^{2}+{Y}^{2}+{Z}^{2}}$$

where rv is the resulting vector and X, Y, and Z are the time series from three dimensions.

From the resulting vector, we extracted three features: (i) RMS amplitude, which was calculated according to Eq. 2; (ii) peak power; and (iii) peak frequency of the frequency spectrum, which were obtained after a fast Fourier transform of the resulting vector.

2 $$RMS=\sqrt{\frac{1}{n}} \sum_{i=1}^{n}{v}_{i}^{2}$$

**Fig. 2:**
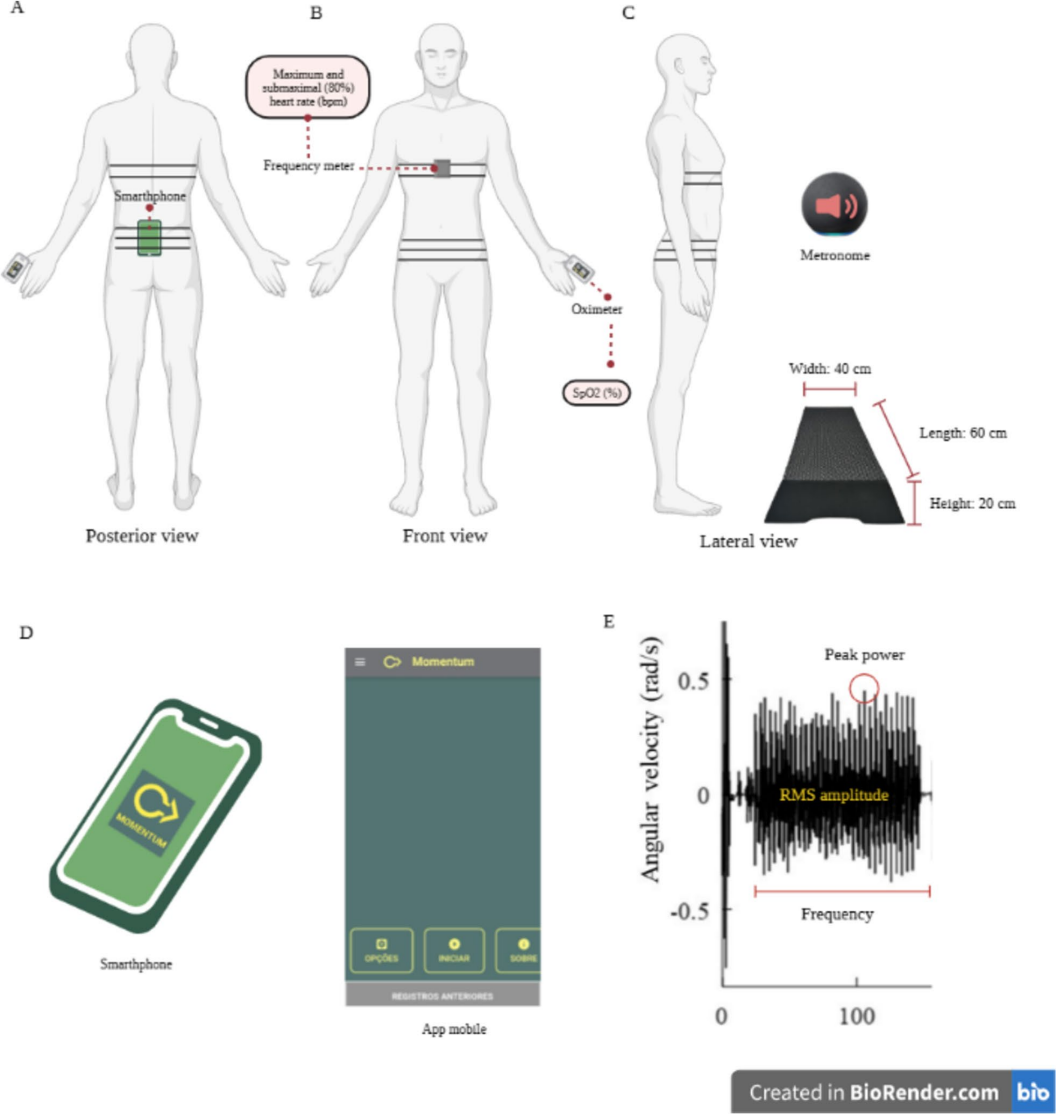
Patient monitoring (**A**-**B**), representative schematic of test performance (**C**), Momentum application interface (**D**), and gyroscope data obtained during testing (**E**)

Figure 2 summarizes the procedures administered to patients and the performance results from the CST.

### Statistical analysis

Descriptive statistical analysis was used to calculate frequencies (absolute and relative (%)), means with standard deviation (parametric) for the clinical and anthropometric variables, and medians with interquartile ranges (IQR) (nonparametric) for the gyroscope variables. The Kolmogorov-Smirnov test was used to assess the normality of the data. The Friedman test (nonparametric) was applied to assess differences (with respect to the gyroscope variables: RMS Amplitude, Frequency, and Peak Power) between levels of the Chester Step Test (CST).

To assess the predictive value of CST in relation to postoperative length of stay, multivariate linear regression analysis was performed including data from the CST level, gyroscope variables (RMS amplitude, frequency, and peak power), work, and VO2max. All statistical analyses were performed using the Statistical Package for the Social Sciences software (SPSS for Windows, v21.0; IBM) with a significance level (α) of 0.05.

## Results

A cohort of 51 patients undergoing abdominopelvic oncologic surgery was evaluated preoperatively and followed up for 30 days postoperatively. All patients met stage 1, whereas 45 (88%) patients met stage 2, 31 (61%) patients met stage 3, 24 (47%) patients met stage 4, and 14 (27%) patients met stage 5 based on test criteria and exercise capacity. The incidence of comorbidities was 27.5% (*n*=14) for hypertension, 11.8% (*n*=6) for diabetes, 15.7% (*n*=8) for obesity, and 5.9% (*n*=3) for heart disease. Smoking and alcoholism were present in 13.7% (*n*=7) and 19.6% (*n*=10) of patients, respectively, with 35.3% (*n*=18) of patients being former smokers and 54.9% (*n*=28) being former alcoholics. Diagnostic hypotheses included gastric cancer (*n*=15; 29.4%), colon or rectal cancer (*n*=10; 19.6%), uterine cancer (*n*=5; 9.8%), and others. The most common oncologic surgeries performed were total and subtotal gastrectomy (*n*=7; 13.7%), hysterectomy (*n*=5; 9.8%), and colon or rectal amputations (n=3; 3.9%). The incidence of ICU admission was 37% (*n*=19), with 11.8% (*n*=6) of these patients requiring invasive mechanical ventilation. The average postoperative hospital stay was 6.36 days, the duration of oncologic diagnosis was 7.91 months, the operative time was 5.7 hours, and three deaths were recorded within the 30-day postoperative period. Table 1 provides a summary description of the sociodemographic and clinical characteristics of the patients.

**Table 1:** Descriptive statistics of patients who underwent abdominopelvic surgeries

**Variable**	***n*** **= 51 (%)**
**Social variables**	
**Sex (%)**	
Female	21 (41.2%)
Male	30 (58.8%)
**Age (years)**	55.71 ± 15.82
**Skin color (%)**	
White	11 (21.6%)
Brown	29 (56.9%)
Black	11 (21.6%)
**Education**	
No schooling	6 (11.8%)
Elementary school	31 (60.8%)
Secondary school	10 (19.6%)
Higher education	4 (7.8%)
**Marital status (%)**	
Single	25 (49%)
Married	20 (39.2%)
Widower	3 (5.9%)
Judicially separated or divorced	3 (5.9%)
**Family income (%)**	
No income	2 (3.9%)
< 1 minimum wage	23 (45.1%)
> 1 minimum wage	26 (51%)
**Anthropometric variables**	
Height (m)	1.62 ± 0.08
Weight (kg)	66.04 ± 16.67
BMI (kg/m^2^)	24.76 ± 5.40
**Clinical variables**	
Postoperative hospital stay (days)	6.36 ± 4.20
Diagnosis time (months)	7.91 ± 7.38
Death (%)	3 (6%)

This section may be divided by subheadings. It should provide a concise and precise description of the experimental results, their interpretation, as well as the experimental conclusions that can be drawn.

### CST results

All participants completed 1 level, 45 participants completed 2 levels, 31 participants completed 3 levels, 24 participants completed 4 levels, and 14 participants completed 5 levels.

The work resulted in a mean value of 486.05 ± 127.32 watts for the study participants.

VO2max calculations were performed for 32 participants who were able to complete more than two levels of the test. The mean value for VO2máx consumption was 51.70 ± 11.74 ml/kg/min.

Inertial measurements to quantify physical activity during CST

**Fig. 3:**
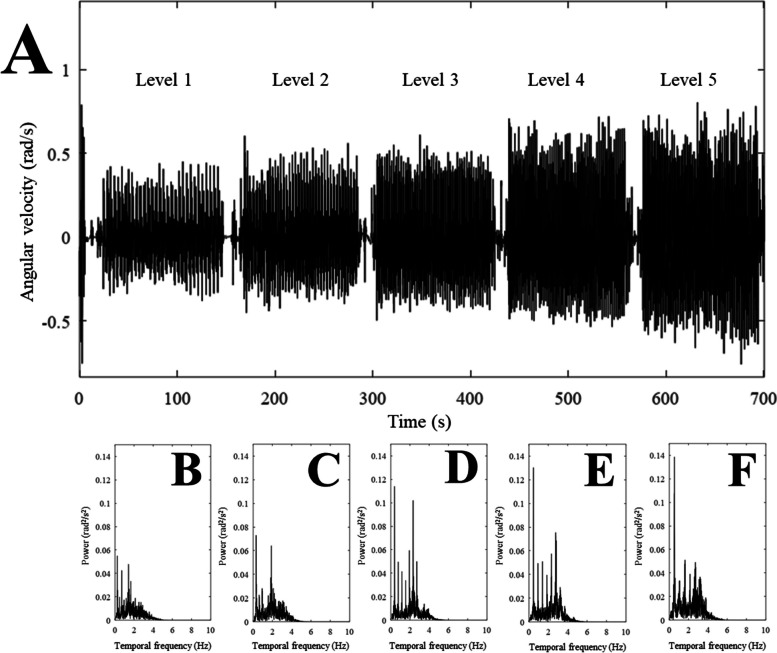
Motion analysis by Octave software recorded by Momentum application gyroscope in the different levels (**A**). Peak power in the levels 1 - 5 (**B** - **F**)

Figure 3 shows inertial recordings (A, time domain; B-F, frequency domain) of a representative participant during five levels of CST. It is possible to visualize differences in RMS amplitude, frequency, and peak power distribution of the inertial series. Table 2 shows the mean values of the variables and the recordings for the patients who completed the different levels.

**Table 2:** Mean values (standard deviation) of inertial parameters of patients who completed the five different levels

**Parameters**	**Values**
RMS amplitude (rad/s)	
1	0.15 (0.59)
2	0.20 (0.71)
3	0.22 (0.92)
4	0.28 (1.03)
5	0.28 (1.03)
Frequency (Hz)	
1	0.23 (0.03)
2	0.30 (0.06)
3	0.37 (0.04)
4	0.42 (0.07)
5	0.51 (0.12)
Peak power (rad^2^/s^2^)	
1	0.07 (0.28)
2	0.08 (0.42)
3	0.12 (0.36)
4	0.15 (0.43)
5	0.13 (0.30)

Root mean square (RMS)

With respect to the gyroscope variables, RMS amplitude (rad/s) (χ^2^ = 54.51; *p* < 0.0001) and frequency (Hz) (χ^2^ = 34.05; *p* < 0.0001) in the levels of CST with increasing progression (Table 2). Peak power (rad2/s2) increased with progression to stage 4 of CST, whereas it decreased at stage 5 (χ^2^ = 26.80; *p* < 0.0001).

### Prediction of length of hospital stay

The relationship between the variables of CST (gyroscope, work, VO2máx, and levels 1-5) and the duration of postoperative hospital stay was analyzed. Levels 1, 3, and 4 showed significance (*p* < 0.05), and the model summary of the final model is shown in Table 2. However, before building the multiple linear regression model, the hypotheses were tested. The result of the Durbin-Watson test was (level 1: 2.13; level 3: 1.92; level 4: 1.93) and was within the acceptable range of [1.5; 2.5] to demonstrate the independence of the residuals.

**Fig. 4:**
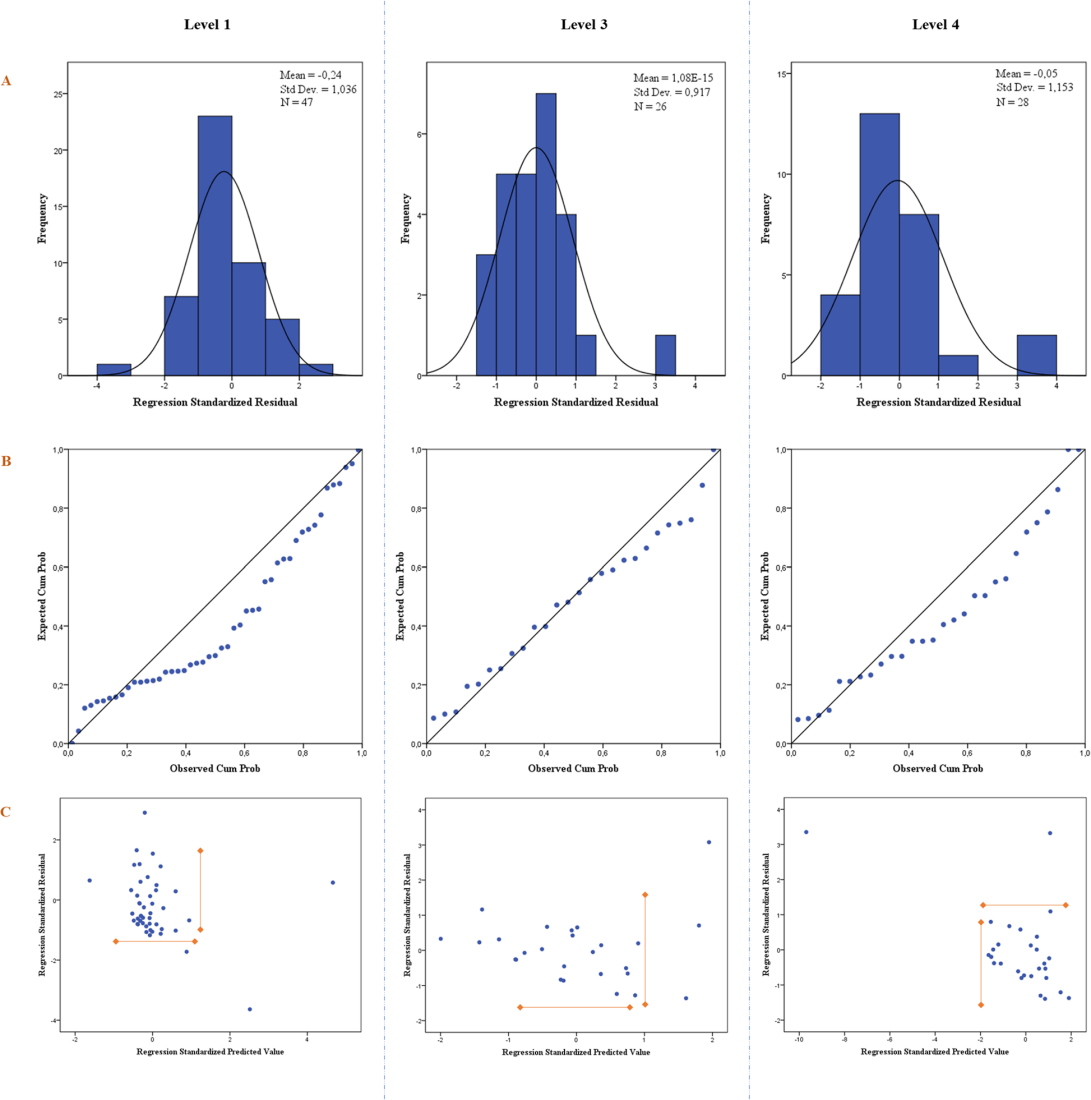
Gaussian distribution (**A**), normal P-P plot of standardized residuals (**B**), and plot of "standardized residuals" against "standardized predicted value" (**C**) for the different levels (1, 3, and 4) in CST

Figure 4 shows the Gaussian distribution (Fig. 4A) and the P-P plot (Fig. 4B), in which a comparison of the "observed probability" with the "expected probability" is used to test the normal distribution of the residuals in the different levels. As can be seen in Fig. 4A, the plots show a parametric distribution, and in Fig. 4B the points are quite close to the line. There are a few outliers, but they have been shown not to affect the quality of the coefficient estimates. The Cook distance (level 1: 0.15 ± 0.63; level 3: 0.09 ± 0.21; level 4: 0.25 ± 1.02) was less than 1 for each observation, so there were no outliers in the data set that negatively affected the estimation of the coefficients. In fact, Cook's distance was calculated for each point, and the mean was well below the required threshold of 1, as shown previously. Figure 4C shows the plot of the "standardized residuals" against the "standardized predicted value" to verify that the variance of the residuals was constant. The variance of the residuals was constant across the predicted values.

Successively, the values of VIF and tolerance obtained for each independent variable were tested, and the variable energy (levels 3 and 4) was excluded from the final model. The values of VIF (< 10) and tolerance (> 0.2) of the final model are shown in Table 4, so the absence of multicollinearity was checked.

The correlation coefficient (R) was high for stage 1 (*R* > 0.66) and moderate for levels 3 and 4 (R: 0.33 to 0.66), so it can be considered as a good model to represent the problem (Table 3).

**Table 3:** Model summary and Fisher's exact test (D) of the final model for the different levels (1, 3, and 4) in CST

**Model**	**R**	**R**^**2**^	**Adjusted R**^**2**^	**Std. Error of the Estimate**	**Sum of squares**	**Degrees of freedom**	**Mean square**	**F**	***p*-value**
*Level 1*									
Regression	0.801	0.641	0.560	5.381	1138.592	5	227.718	0.003	0.956
Residue					637.122	22	28.960		
Total					1775.714	27			
*Level 3*									
Regression	0.584	0.341	0.215	7.289	576.236	4	144.059	0.008	0.932
Residue					1115.803	21	53.133		
Total					1692.038	25			
*Level 4*									
Regression	0.613	0.375	0.219	7.681	567.296	4	141.824	0.415	0.529
Residue					943.942	16	58.996		
Total					1511.238	20			

Table 4 shows the coefficients of the model for levels 1, 3, and 4 of CST and the results of the t-test used to examine the significance of the regression coefficients (β).

**Table 4:** Relationship between the variables CST (gyroscope, work, steps, and VO2máx) and length of postoperative hospital stay

				**Collinearity statistics**	
**Variables**	**Standardized Coefficients β**	**t**	***p*-Value**	**Tolerance**	**VIF**
*Level 1*					
Work	0.230	1.505	0.147	0.697	1.435
RMS Amplitude 1	-1.119	-3.441	0.002*	0.154	6.481
Peak Frequency 1	0.165	1.118	0.276	0.752	1.329
Peak Power 1	1.362	5.033	<0.0001*	0.223	4.494
VO_2max_	-0.046	-0.261	0.796	0.531	1.884
*Level 3*					
Work	0.693	2.733	0.012*	0.488	2.047
RMS Amplitude 3	0.507	1.908	0.070	0.444	2.252
Level	-0.422	-1.783	0.089	0.561	1.781
VO_2max_	-0.650	-2.639	0.015*	0.517	1.934
*Level 4*					
Work	0.468	1.973	0.066	0.693	1.443
RMS Amplitude 4	0.312	1.288	0.216	0.666	1.501
Level	-0.322	-1.507	0.151	0.856	1.168
VO_2max_	-0.435	-2.127	0.049*	0.936	1.069

^*^ *p* < 0.05

Table 4 shows that levels 1, 3, and 4 of CST are predictors of postoperative hospital stay in patients undergoing abdominopelvic surgeries. The *p*-value was less than 0.05 for RMS amplitude and peak power of giroscopy at level 1; work and VO2máx at level 3 (25 steps/min) and VO2máx at level 4 (30 steps/min). Levels 2 and 5 showed no statistical significance in the constructed models.

## Discussion

According to our findings, the accuracy of detecting changes for predicting an unfavorable outcome in patients who met CST stage 1 was higher with the gyroscope features (i.e., root mean square amplitude and peak energy) compared to VO2max. However, the latter was not effective in predicting prolonged hospitalization until level 3 of the test, indicating limited applicability for patients with lower exercise capacity. Moreover, the study showed that the instrument had low accuracy in determining the patient's true functional capacity.

The use of inertial sensors in smartphones for patient monitoring during exercise and rehabilitation has become prevalent [21]. Numerous studies have reported the high reliability of measurements obtained using smartphone gyroscopes, which have been identified as a precise sensor for evaluating postural stability [22–24], fall risk in the elderly [25, 26], rehabilitation of patients with vertigo and balance disorders [21], stroke patients [27], individuals with Parkinson's disease [28], multiple sclerosis [29], and other related medical conditions..

One systematic review examined the use of inertial sensors in smartphones, including gyroscopes, to measure the various parameters of the Timed-Up-and-Go (TUG) Mobility Test to detect different patterns associated with different diseases and identify future risk situations [30]. In another study, aspects of mobility that are not routinely assessed, including trunk angular velocity, which reflects trunk rotation during turning and the transition from sitting to standing, were found to be independent predictors of disability and mortality in the elderly [31]. This demonstrates the wide applicability of the smartphone gyroscope as an alternative to modern, expensive devices.

Individuals who are hospitalized typically already have impaired functioning prior to their hospitalization, either due to health status, age, or both [32, 33]. This makes hospitalization an important determinant of functional decline, independent of physical frailty [34, 35]. Because functional capacity is a direct determinant of mobility, mobility rates are thought to tend to decline after hospitalization, with low mobility being a predictor of poor hospital outcomes after discharge [36].

Cancer patients often experience loss of muscle mass and altered muscle function, which is exacerbated by the toxic effects of cancer therapies [37]. These impairments in mobility have been associated with treatment-related symptoms such as abdominal swelling, fatigue, loss of appetite, numbness, tingling, and pain [38]. Persistent mobility impairments have also been reported following neurotoxic chemotherapy, such as peripheral neuropathy [39]. These symptoms represent potential targets for intervention in preserving mobility and physical function in cancer survivors. Extensive physical deterioration resulting from cancer, treatment, and hospitalization can contribute to decreased mobility and longer hospital stays, particularly in patients who progress to CST stage 1, which often limits progression to other levels.

Although the CST-score is less commonly used today because VO2máx is preferred as a better predictor of test results [14, 40], our results showed that peak power and RMS amplitude expended at level 1 and work done at level 3 were predictors of longer postoperative hospital stay. Energy can be defined as anything that can be converted into work or heat, and work is a measure of the energy transferred by the application of a force along a displacement [41]. Therefore, assuming that mechanical energy is the ability to do work, the gyroscope (peak power) was sensitive in detecting energy expenditure at level 1, while work was detected only at level 3, showing that patients with lower mobility or higher values of RMS amplitude had higher energy expenditure.

Cancer cells generally exhibit increased aerobic glycolysis to meet the increased metabolic demands of malignancy [42]. The development of the disease itself demands a lot of energy from the body, and any activity that has a certain intensity also requires an increased energy expenditure. However, energy expenditure is affected by cancer in a heterogeneous manner and may have components of hypo- and hypermetabolism depending on tumor type, cancer stage, and treatment modality [43]. Hypermetabolism has been associated with neoplasms of the gastrointestinal tract, including gastric, esophageal, and colon cancers, as well as solid tumors and gynecologic neoplasms, such as ovarian and cervical cancers, and is even associated with postoperative recurrence of pelvic lesions [44]. This correlates with the profile of patients studied who underwent abdominopelvic surgery.

The metabolic changes induced by disease and treatment, combined with decreased food intake and oxygen availability, form the multifactorial basis of cancer-related malnutrition or cachexia [45]. The energy we derive from food depends on oxygen for the process of nutrient combustion. The oxidation of nutrients produces species with more energetically stable chemical bonds that allow a negative energy balance between products and reactants, which is responsible for the release of energy, either in calorimeters or in the metabolism of the human body [46]. Thus, in cancer patients with restricted diets and lower aerobic fitness, the higher energy expenditure becomes more evident.

The CST is a valid and acceptable means of estimating VO2max in the general adult population, making it a suitable tool for tracking changes in cardiorespiratory fitness due to its high test-retest reliability [14]. The CST expresses the functional capacity of the individual and correlates well with other field tests such as the shuttle test and the 6-minute walk test [13, 47]. Patients with better functional capacity, particularly from stage 3, tend to have shorter hospital stays.

Elderly patients, women, and patients with advanced tumor stages tend to have lower cardiorespiratory fitness scores [40]. In one study, length of hospital stay correlated with the number of steps in patients with acute lung disease, with no differences between the CST and Modified Incremental Step Test (MIST) protocols [48].

The literature is limited regarding the use of CST in preoperative patients, however, the use of other field tests such as the 6MWT has been well studied in this population, including gastroesophageal [49] abdominal, and pelvic [50] cancers. A significant, positive, and high correlation was found between CST and 6MWT, which can serve as a reliable surrogate for measurement properties [13]. However, the option to study CST was chosen due to its better applicability.

Thus, it is observed that the gyroscope features were more sensitive in detecting reduced functional capacity of decline in mobility, higher energy expenditure, in addition, VO2máx remains a good predictor of outcomes in inpatient oncology patients. Therefore, the practical clinical applicability of the study involves implementation in health services, as a quaternary prevention resource [51], since physical capacity can be assessed prior to surgical intervention and if there is a risk of a worse prognosis, the patient can receive intensive preparatory treatment in the pre-operative period to carry out the surgery with a greater chance of success.

## Limitations

A substantial number of patients could not be studied because of deterioration of their physical condition, progression of the disease, or the effects of chemotherapy that prevented or made impossible the performance of the test.

## Conclusions

Therefore, the length of hospital stay was longer 30 days after surgery when patients had lower RMS amplitude and higher peak energy at level 1 of the test. In addition, work performance increased as the test progressed from level 3. High VO2máx appears as a predictor of length of stay for those who completed levels 3 and 4 of the test.

Thus, use of the gyroscope was more accurate in detecting changes in RMS amplitude and peak power that would predict a less favorable outcome for those who met stage 1 of CST. VO2max was not able to predict prolonged postoperative hospital stay until level 3 of the test, limiting the tool for patients with lower exercise capacity. Work was less accurate in detecting the actual functional capacity of the patient.

## Data Availability

Please contact the corresponding author.
